# Healthcare wastewater surveillance: methodological considerations for sampling, feasibility, and implementation

**DOI:** 10.2166/wh.2025.167

**Published:** 2025-12-22

**Authors:** Angela Coulliette-Salmond, Florence Whitehill, Amanda K. Lyons, Bethelhem Abera, Colin Adler, Maroya Spalding Walters, Magdalena Medrzycki, Christine Ganim, Mariya Campbell, Michael Y. Lin, Rachel S. Poretsky, Adam Horton, Jennifer Weidhaas, James VanDerslice, L. Scott Benson, Erin M. Driver, Rolf U. Halden, Kerry A. Hamilton, Margaret Williams

**Affiliations:** a CDC, Division of Healthcare Quality Promotion, Atlanta, GA, USA; b U.S. Public Health Service, Rockville, MD, USA; c Rush University Medical Center, Chicago, IL, USA; d University of Illinois-Chicago, Chicago, IL, USA; e University of Utah, Salt Lake City, UT, USA; f The Biodesign Institute, Arizona State University, Tempe, AZ, USA; g School of Sustainable Engineering and the Built Environment, Tempe, AZ, USA

**Keywords:** facility-level, feasibility, healthcare, wastewater surveillance

## Abstract

Wastewater surveillance (WWS) at healthcare facilities is a nascent field with knowledge gaps in the feasibility of conducting such surveillance at this specialized facility type, and for how to best implement and interpret wastewater (WW) data. WWS was piloted at skilled nursing facilities, including conducting tracer studies, optimization of a low-flow strainer for autosampler WW collection, and preliminary testing of a WW access survey. An expanded WW access survey with collaboration from additional partners was distributed to 16 post-acute/long-term care facilities. The lessons learned obtained through the pilot ‘use cases’ demonstrated minimal clogging and consistent collection of WW using a low-flow strainer (±0.36 L) and tracer studies highlighted the importance to confirm facility effluent source with an average visual dye detection between 1.5 and 2.5 min from the toilet flush. The expanded WW access survey assessed the feasibility of WWS regarding physical onsite manhole access, safety aspects, effluent flow, and other factors, where 75% (12 of 16) of surveyed facilities demonstrated feasibility. Healthcare facility-level WWS includes specialized methodological approaches prior to implementation to achieve the intended public health impact. These considerations support the continued overall goal of detecting emerging biological public health threats at healthcare facilities using WWS.

## Graphical Abstract

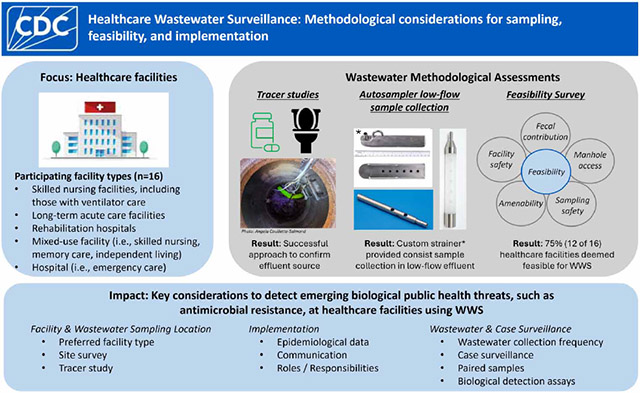

## INTRODUCTION

Facility-level wastewater surveillance (WWS) has been explored, particularly during the severe acute respiratory syndrome coronavirus 2 (SARS-CoV-2) pandemic, with studies that focused on the association between wastewater (WW) results and COVID-19 cases (Acosta et al. 2021; Gamage et al. 2024; Keck et al. 2024; Santiago et al. 2025). Recently, carbapenem-resistant organisms and carbapenem-producing genes are being explored in hospital and healthcare WW on understanding how antimicrobial resistance from hospitals/healthcare contribute to community watershed resistance, what the bacteria and gene signals are in the facility-level effluent, and if there’s potential utility of facility-level WWS to indicate a clinical presence (Hassoun-Kheir et al. 2020; Hassoun-Kheir et al. 2025; Krul et al. 2025; Lenz et al. 2025; Poretsky et al. 2025; Santiago et al. 2025; Silvester et al. 2025). These topics are foundational, yet publications describing factors to consider before implementing healthcare-focused facility-level WWS with the intention to correlate clinical prevalence of non-viral, public health threats are limited. The type of healthcare facility and targets for meaningful public health gains, field and building assessment regarding the feasibility of WW sampling, as well as the inclusion of multidisciplinary stakeholders prior to initiating WWS, impact the outcome strength and contribution toward a utility determination of healthcare facility WWS.

Healthcare facilities with high-acuity patients could benefit from WWS for biological public health threats, such as the listed ‘urgent threats’ of carbapenem-resistant Enterobacterales (CRE) and *Candida auris* (CDC 2019), to potentially limit regional spread and protect the larger surrounding community with earlier detection. Facilities that care for high-acuity patients with long lengths of stay are considered ‘influential facilities’ with a disproportionate impact on regional transmission, where long lengths of stay are typically defined as >30 days (CMS 2016a, 2016b; AHA 2019). Models demonstrate that earlier detection with implementation of targeted infection control practices would reduce regional spread (Real 2004; Smith et al. 2004; Slayton et al. 2015; CDC 2022; Cincotta et al. 2022). Healthcare WWS is a notable early approach that has potential utility to support clinical screening practices and inform public health stakeholders on circulating and emerging antimicrobial resistant (AR) organisms, including *C. auris*. Thereby, the utility of WWS for the proactive detection of healthcare AR threats at the healthcare facility has come to the forefront (Foxman et al. 2023; NAS 2024).

Before initiating healthcare facility WWS, several engineering and field aspects should also be considered. The facility building and plumbing, for example, likely did not consider WWS in design decisions and may not include a sewer manhole on the property. Historical knowledge and building blueprints are often unavailable prior to WWS implementation, thereby leaving the confirmation of the facility population captured by WWS uncertain. Additionally, WW sampling approaches have not been fully optimized at the healthcare facility level to address intermittent, low-flow, and healthcare-specific debris. Particularly for autosamplers, there are unforeseen complications at healthcare facilities due to the autosampler design intended for a fully submerged vacuum line. Combined with the nuances above, the safety aspects of collecting WW and the overall feasibility of conducting WWS at a healthcare facility are additional considerations.

Determining the utility of healthcare WWS is an important initial phase prior to utilizing these data to inform public health measures. It is critical to develop robust study designs that pair WW data with epidemiological and patient screening data from the healthcare facility, enabling accurate correlation analyses of WW signals with disease dynamics. Collecting these data is most easily accomplished after laying the groundwork for strong communication and collaboration between the WWS team, the facility, and the jurisdictional health department. With the long-term goal of establishing a WWS framework specific to healthcare facilities that enables future clinical data correlations for AR organisms or emerging pathogens of public health concern, we present lessons learned and key methodological considerations based on data collected in collaboration with 16 U.S. post-acute/long-term care facilities from 2022 to 2024. These insights serve as initial steps toward this goal to assist fellow stakeholders.

## METHODS

This effort met the US Centers for Disease Control and Prevention (CDC) safety policies and recommendations in the CDC/NIH *Biosafety in Microbiological and Biomedical Laboratories* (Meechan & Potts 2020). This activity was reviewed by CDC, deemed research not involving human subjects, and was conducted consistent with applicable federal law and CDC policy^§^.

### Healthcare WWS partners

The Healthcare-Wastewater AR Network (H-WARN) program within the Division of Healthcare Quality Promotion at CDC (Atlanta, GA) collaborated with the Georgia (GA) Department of Public Health (GDPH), Rush University Medical Center in partnership with the University of Illinois – Chicago, the University of Utah, and Arizona (AZ) State University (ASU) to conduct WWS at post-acute/long-term care facilities. In this paper, the seven GA facilities were identified as Facility A to G, the five Illinois (IL) facilities were identified as Facility H to L, the Utah (UT) and Texas (TX) facilities were identified as Facility M and N, respectively, and the two AZ facilities were identified as Facility O and P. Types of care provided by each facility are described in Table 1 and individual state partnerships and facility selection are described in Supplemental 1 (S1).

### Lessons learned – tracer studies and strainer assessment

#### Facility descriptions:

Three skilled nursing facilities (A, F, and G) in the Atlanta metro area (GA) participated in the tracer studies, while the strainer assessment was conducted only at Facility A. The tracer studies and strainer assessment were piloted, preliminary efforts, as ‘use cases’ to drive best practices moving forward and to guide future partners. While a limited number of facilities and sample sizes were included, the presented data were a significant driver in next-step decisions. The investigative approach is shared so others can implement similar preliminary efforts prior to a full-scale WWS study.

#### Tracer studies:

The two-part tracer study included a fluorescent tracer dye (Bright Dyes^®^ FLT yellow/green tablet; Kingscote Chemicals, Miamisburg, OH) followed by a biological surrogate on a subsequent day, which was heat-inactivated (60 ± 2 °C for 40 min) Inforce^®^ 3 Respiratory lyophilized calf vaccine (Parsippany, New Jersey; Zoetis) at an eluted final concentration of 10^6^ to 10^9^ copies per 50 mL (biological surrogate target: bovine respiratory syncytial virus). The tracer study was used to gauge WW residence time (i.e., the amount of time WW spends in pipes between toilet and collection/sampling points) after a toilet flush and confirm that the selected manholes captured the intended population at Facilities A, F, and G. Methods are further described in S1.

#### Strainer assessment:

Three strainers compatible for use with the Teledyne ISCO Avalanche Autosampler (Lincoln, NE) were evaluated at Facility A with the aim of consistent collection of the desired WW sample volume (Supplemental Figure 1). The Standard Weighted Polypropylene Strainer (‘Standard’, *n* = 9 volume comparisons between ‘expected’ and ‘collected’; Lincoln, NE; Teledyne ISCO), Low-Flow Stainless Steel Strainer (‘Low Flow’, *n* = 7; Lincoln, NE; Teledyne ISCO), and a custom fabricated strainer (‘Custom’, *n* = 9; Atlanta, GA; CDC) were deployed, and ‘expected’ and ‘collected’ sample volumes were recorded. The autosampler, using either a flow or time-based program, aimed for a total ‘expected’ sample volume of 0.8 L for the 24 h composite collection time. Statistical analysis and figure creation used GraphPad version 10.2.0 (Boston, MA), where the absolute values of the ‘expected’ and ‘collected’ differences were used to determine the significance between the groups with Kruskal–Wallis and Dunn’s *post hoc* tests (*p* < 0.05). See Supplemental Figure 1 for strainer visuals and design differences; the Custom strainer technical drawing in Supplemental 2.

### WWS feasibility assessment

#### Pilot survey:

A structured survey specific to WWS at the facility level had not been published or readily available when WWS efforts were initiated at the healthcare facility-level during the COVID-19 pandemic. A structured survey was developed for documentation of clear criteria for facility-level WWS. The lead author designed the survey based on WW and environmental surveillance field experience with ‘yes’ or ‘no’ questions and free text fields to enter additional details. The main goal of the survey was to determine if it was feasible for a WWS project to take place at a given facility. The survey was structured as eight yes/no questions on the following topics: physical access for WW effluent on the designated property – is there a sewer manhole on the property?; safety for those occupying and/or visiting the facility during purposed WWS if WW is being collected – will facility staff, visitors, and/or patients be at risk for exposure to WW or injury due to the equipment?; safety for those sampling WW at the facility – does the manhole location put WW collectors at risk due to being in a high traffic area, etc.?; facility amenability to WWS – does the facility understand what WWS will physically look like at their facility and open to the duration of the surveillance?; and additional WW feasibility considerations (i.e., outlet nearby for autosampler, manhole condition, proper effluent flow).

The survey was completed during initial facility site visits by H-WARN staff through visual observations and if needed, follow-up verbal conversations with relevant staff. This was administered onsite at seven GA post-acute care facilities (Facilities A-G) between 2021 and 2022, where the primary respondents included facility administrators, grounds maintenance staff, directors, nurses, and infection control specialists (Table 1). The final determination of feasibility was considered ‘no’ if there was any safety risk, poor manhole condition, or inadequate effluent flow.

#### Expanded pilot survey:

Partners in IL and UT collaborated with H-WARN during four virtual discussions between March 2023 and July 2023 to modify the pilot survey’s existing data elements and incorporate new elements that improved the survey’s ability to assess the feasibility of performing WWS at a facility. Modifications included adapting the format of the survey for improved entry into a digital database. New questions expanded on the willingness of healthcare facilities to participate in WWS to better understand the likelihood of and barriers to participation. Additional questions regarding other potential influxes into the WW effluent, such as grease traps, that could interfere with fecal signals, as well as a question regarding clean-outs as a back-up for passive sampling, were also included for a broader perspective of healthcare facility-level infrastructure.

The 28-question expanded pilot survey was administered during in-person site visits at 9 post-acute care facilities in IL (5), UT (1), TX (1), and AZ (2) that included long-term acute care hospitals (LTACHs), skilled nursing facilities (SNFs), ventilator-capable SNFs (vSNFs), as well as a rehabilitation hospital (RH), mixed-use facility, and general hospital. The surveys’ new sections covered administrative elements, willingness to participate, WW access, grease traps/drainage lines, and plumbing aspects. The expanded pilot survey questions are provided in Supplemental 3 (S3).

#### Feasibility:

Feasibility was determined by three main factors: facility-level WW access availability, WW access practicality, and facility administration amenability. WW access availability incorporated understanding if a sewer manhole was on facility property, whether the access point WW captured the target population, and if the condition of the WW access point and piping were compatible for safe collection and use of sampling equipment. WW access practicality considered whether there was ample room for sampling personnel and the required equipment, the location was safe for sampling with respect to the sampling personnel, the facility residents/patients, visitors, and healthcare personnel, and that the regular function of the facility was not interrupted. The overall facility amenability, meaning a willingness to participate at both facility-level and corporate levels for the duration of the planned WWS, includes considering consistent point(s) of contact (POC(s)), weekly questionnaires completion, and communication to explain the study purpose for the impacted audience (i.e., residents, visitors, staff).

### Key considerations

The key considerations were grouped into three categories: ‘Facility and Wastewater Sampling Location’, ‘Implementation’, and ‘Wastewater and Case Surveillance’. These categories highlight processes applied during healthcare WWS in the COVID-19 pandemic in collaboration with GA DPH (2021 to 2023) to achieve the intended public health impact of WWS and examine WWS feasibility at the healthcare facility level. The considerations were then applied and substantiated during AR WWS efforts with IL, UT, and AZ (2022 to 2024). Key considerations listed in Box 1 were identified as fundamental aspects of a healthcare WWS workflow that is able to connect WW data to facility-level clinical data and involve relevant stakeholders and data elements for full interpretation. A consensus of the presented considerations was reached among participating healthcare facilities, funded partners, health departments, and additional subject matter experts regarding AR organisms, clinical screening, WWS, laboratory networks, and healthcare systems.

## RESULTS AND DISCUSSION

### Lessons learned – tracer studies and strainer assessment

The tracer study was conducted at Facilities A, F, and G, while the strainer assessment was conducted at Facility A only. Facility A and Facility F were free-standing, multilevel buildings, and Facility G was a free-standing building with one level.

#### Tracer studies:

The visual dye studies were conducted in toilets on the fifth floor (topmost floor housing residents) of Facility A (*n* = 2 tracer studies) and the ground floor (only floor housing residents) of Facility G (*n* = 1). At Facility F (*n* = 3), the first dye tracer study was unsuccessful on the first and fourth floor bathrooms from the middle of the facility, and was not visually observed at the manhole that the facility believed would capture the intended section of the building. As a result, a thorough walk-through of facility plumbing details, an evaluation of facility maps, and an assessment of manhole locations were conducted. The subsequent tracer study at Facility F involved two ground-floor toilets in separate facility wings, with simultaneous observation at two manholes. This effort was successful and confirmed the proper manholes for WWS in Facility F. Average water use for Facility A (*n* = 9) was 20,472 gal per day (min:15,685, max: 27,702), Facility G (*n* = 8) was 9,054 gal per day (min: 7,646, max: 10,542), and Facility F (*n* = 8) was 20,698 gal per day (min: 19,270, max: 24,536). Overall, the three facilities had visual detection between 1.5 and 2.5 min from toilet flush and dissipation of the dye tracer between 4 and 7 min from initial visual detection (Table 2).

**Figure 1 ∣:**
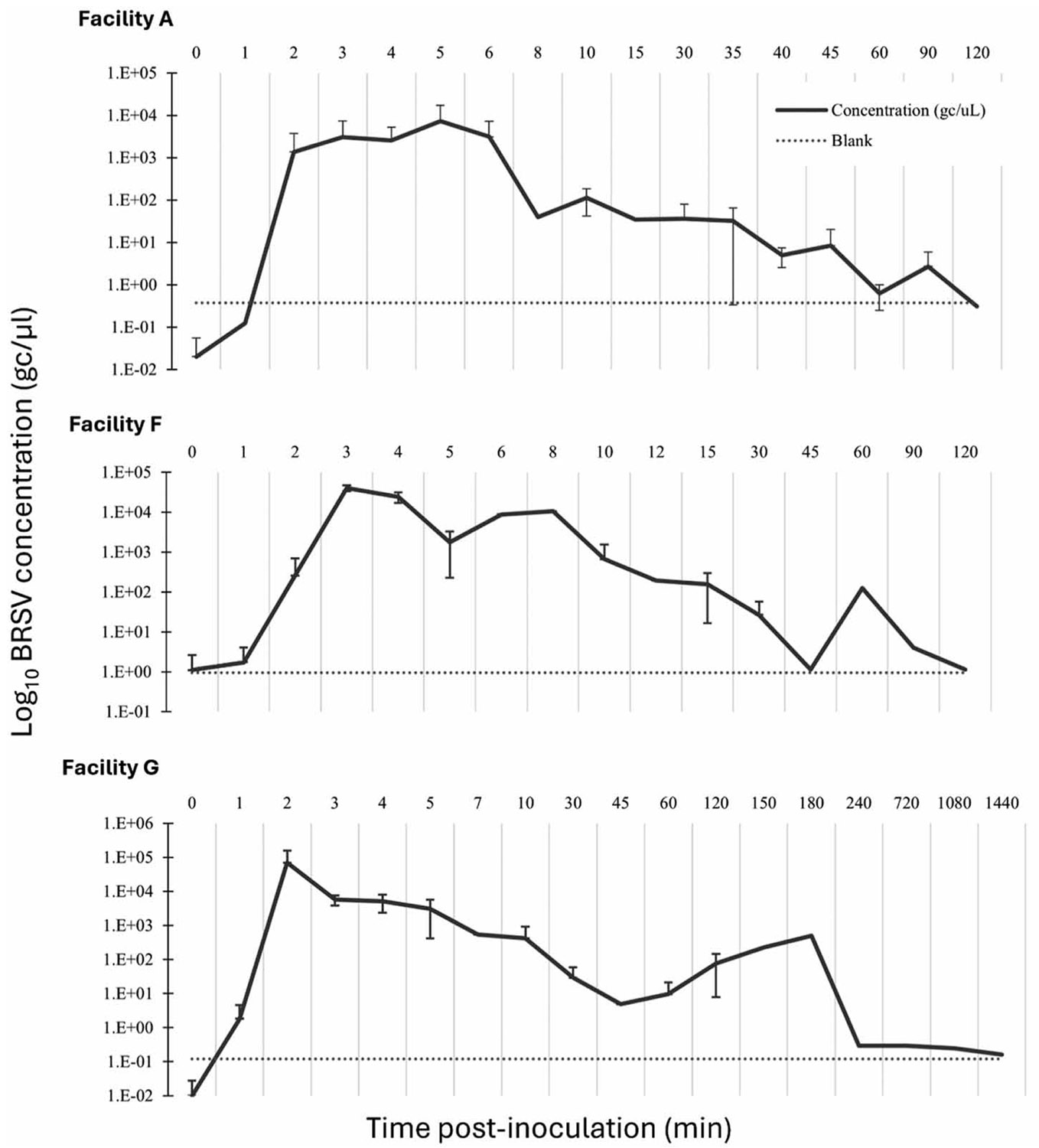
Biological surrogate tracer study concentrations (gc/μl) of heat-inactivated Inforce^®^ 3 Respiratory Vaccine lyophilized calf vaccine with bovine respiratory syncytial virus (BRSV) as the target, post-inoculation to dissipation, for three Georgia skilled nursing facilities (*n* = 3 per facility). Facility A study was conducted from the fifth floor (*n* = 17 time-points), Facility F study was conducted from the first floor (*n* = 16 time-points), and Facility G study was conducted from the first floor (*n* = 19 time-points).

The biological surrogate tracer studies were subsequently performed at Facilities A, F, and G (*n* = 3 per facility) in the same successful locations as the visual dye tracer (Table 2, Figure 1). Facility A (*n* = 17 samples) and F (*n* = 16 samples) studies were conducted over a 120-min time window, while Facility G (*n* = 19 samples) was performed over a 1,440 min (24 h) time window. Initial peak biological surrogate concentrations were detected at 2 min at all facilities with a decreased peak between 10 and 15 min and dissipation time-points ranging from 40 min at Facility A to 240 min at Facility G. At Facility G after the initial decline, there were notable concentration increases between 150 and 180 min (2.23 × 10^2^–4.95 × 10^2^ gc/μl) before continuing to decline and the reason for the extension to 1,440 min. Concentrations over time are shown in Figure 1.

#### Strainer assessment:

**Figure 2 ∣:**
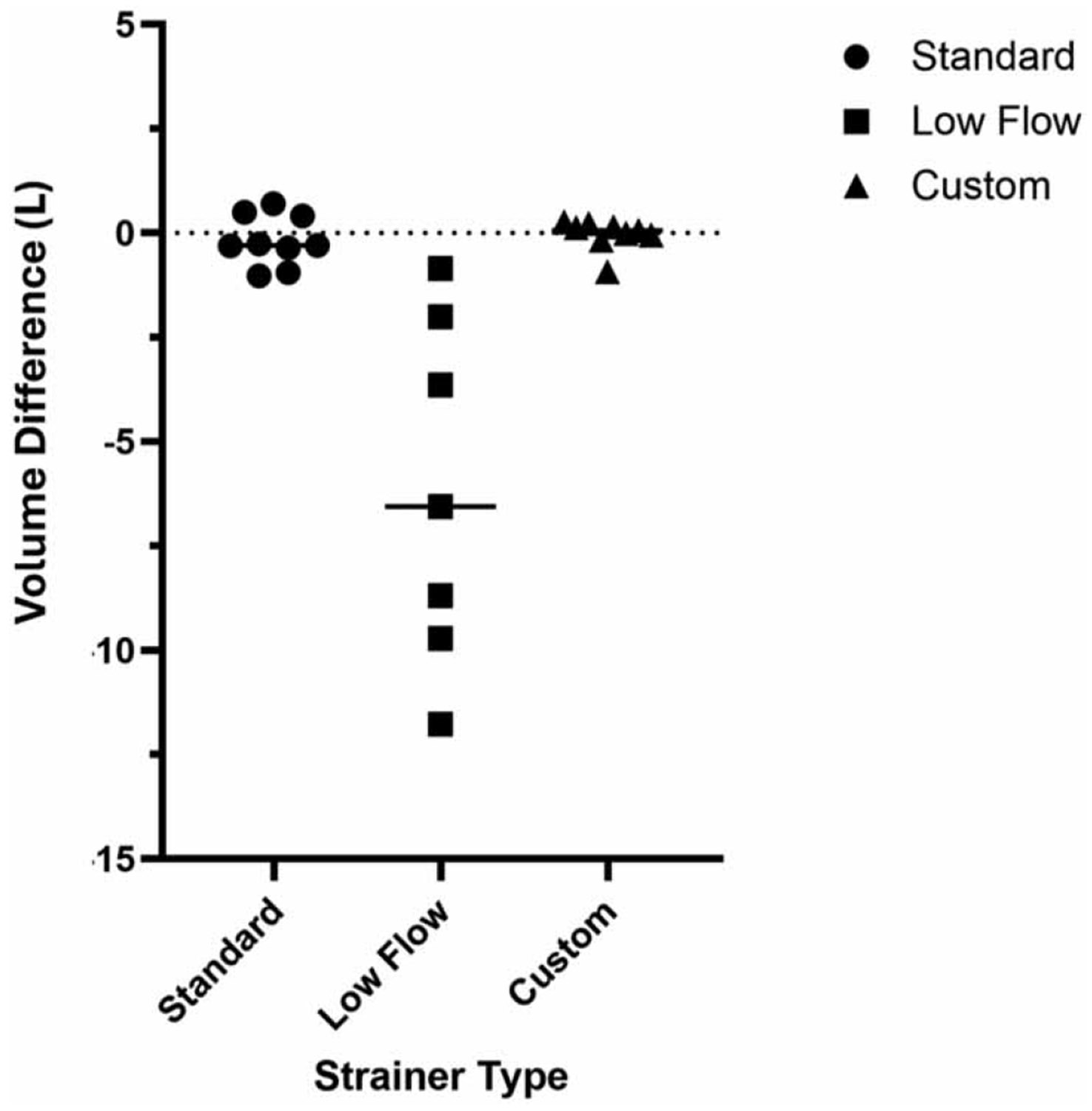
Strainer comparison of the ‘Standard’ (*n* = 9), ‘Low Flow’ (*n* = 7) and ‘Custom’ (*n* = 9) auto sampler compatible strainers for wastewater sampling, where the mean volume differences (expected [0.8 L] versus collected) were −0.18 (±0.61), −6.18 (±4. 14), and −0.04 (±0.36) L, respectively. See Supplemental 1 for strainer photos and Supplemental 2 for the custom strainer technical drawing.

The median flow at the facility during the strainer assessment was 16.9 gal per min (min: 4.1, max: 32.7 gal per min; *n* = 19). However, of note, throughout the assessment for all three strainers, heavy debris impacted the flow meter insert, causing the liquid detector to post errors that no liquid was detected multiple times within the 24h collection period (data not shown). The Standard strainer was deployed for 3 weeks (*n* = 9 volume comparisons between ‘expected’ and ‘collected’), the Low-flow strainer for 2 weeks (*n* = 7), and the Custom strainer for 3 weeks (*n* = 9). Complications with the Standard and Low-flow strainers included the strainer collection holes being blocked by healthcare wipes and the collection holes not being fully immersed, thereby inhibiting WW collection and often creating air bubbles in the line, which caused inaccurate collection. The Custom strainer, noting that healthcare wipes were snared on the strainers’ eye bolt and long barbed hose nipple (see strainer technical drawing, Supplemental 2), remained capable of WW collection due to the design with collection holes exclusively on the bottom (i.e., fully immersed in the low-flow WW) with mini-rails lifting the strainer off the bottom sewer by millimeters, allowing consistent and accurate sample collection (Figure 2). These design differences were reflected in the expected (programed, 0.8 L) versus collected volumes (L), where the volume differences for standard, low-flow, and custom strainers were −0.18 L (±0.61), −6.18 L (±4.14), and −0.04 L (±0.36), respectively. Significant differences calculated using the absolute values of the mean volume differences were demonstrated between all three strainer types (*χ*^2^(2) = 17.8; *p* = 0.0001): standard and low-flow (*p* = 0.0273), standard and custom (*p* = 0.0319), and low-flow and custom (*p* = <0.0001) strainers. The strainer comparison concluded that successful, consistent sampling was achieved using the custom strainer and flow-weighted program set at 25 mL being collected every 700 gal. However, due to challenges using the flow-meter insert, ultimately a time-based program was used for future WWS.

The tracer study revealed inaccurate sewer plumbing knowledge for one of three facilities in GA, emphasizing the importance of confirming a WW effluent source before initiating WWS efforts that will be associated with clinical data. This ‘lesson learned’ was relayed to the Illinois, Utah, and Arizona WWS partners, who successfully conducted visual dye tracer studies to confirm WW collection sites. The four Illinois facilities where the visible dye tracer was conducted (Facility H to K) resulted in the detection time ranging between two to thirteen min, depending on the floor conducted and acknowledging varying census of 28 to 98 patients (two to six min for Facility H – two floors tested, *n* = 3), two to thirteen min for Facility I (*n* = 2) – two floors tested, three min for Facility J (*n* = 2) – two floors tested, and seven to eight min for Facility K (*n* = 2) – two wings tested; census listed for each facility in Table 1). The Utah facility (Facility M, *n* = 2) dye tracer resulted in 5 min detection from the first floor and 30 min from the 4th floor (census of 45). The AZ facility (Facility O) dye tracer resulted in a little over three and a half min from the second and third floors (*n* = 13), respectively (census of 11). Overall, the visible dye tracer importance of conferring the facility population contribution to the effluent was the priority and was successful for all tested facilities. The detection times for the facilities evaluated provide a range of time to expect to see the dye after flush (2 min and up to 30 min).

The strainer assessment, while a small sample size, provided guidance to use the customized strainer for the remainder of the WWS at the three GA skilled nursing facilities to obtain consistent volumes and avoid healthcare debris. Conducting side-by-side comparisons during the COVID-19 pandemic was not a priority at the time, and the design provided is available for fabrication (Supplemental Materials 2). It is important to note that the consistent collection of WW was dependent on the concurrent operation of the flow meter, flow-weighted program, and strainer. The challenges of low-flow conditions, intermittent effluent, and clogging from healthcare debris (i.e., sanitary wipes) highlight the advantages of passive sampling, which would not encounter these technical aspects like an autosampler. Poretsky *et al*. concluded that passive sampling was preferable over using an autosampler at a long-term acute care hospital due to comparable detection of targets, higher recovery in spiked-in controls, and the less expensive nature to scale to multiple facilities (Poretsky 2025 #18). Warren *et al*. additionally found that passive sampling at a rehabilitation hospital was as effective as composite sampling and detected an increased concentration of carbapenemase-producing genes (Warren et al. 2025).

### Feasibility assessment

The facility general descriptions, average census, facility types, and survey results are detailed in Tables 3 and 4, where footnotes provide specifics regarding access points, safety concerns, and extenuating circumstances that would make WWS not feasible. Results per state for the pilot and expanded surveys are detailed in S1.

#### Overall Feasibility (Combined Facilities A – P):

Twelve (75%) of 16 total facilities surveyed (pilot and expanded pilot surveys) determined that WWS was feasible. Of the 16 total healthcare facilities surveyed, 15 (93.8%) had at least one external physical access point with WW access availability, where 10 (66.7%) of the 15 were located on landscape grounds versus a paved area with pedestrian or vehicle traffic. Focusing on the nine facilities included in the expanded pilot survey, only one facility (11%) had a nearby outlet available to power an autosampler. The most common safety concerns centered on the WW access being a manhole located in an area with pedestrian or vehicle traffic, and manhole covers being of atypical size/shape that would interfere with typical WW sampling equipment. Of the eight expanded pilot surveyed facilities with WW access availability (Facility J did not have an onsite WW access point), five (62.5%) had laundry services (two sites had bleach injectors and one had a dedicated bedside commode disinfection room). Facility amenability was noted for all 9 (100%) expanded pilot surveyed facilities. Of note, those conducting WWS (e.g., academia) without established links to public health (i.e., post-acute/long-term care facilities) had more challenges with engagement than the partners who had existing partnerships.

Conducting an onsite, in-person survey to understand the feasibility was vital to incorporate the array of field aspects, safety considerations, and facility willingness. The 75% feasibility determination from 16 facilities in five states provides a starting point for assessing the potential of broader WWS at healthcare facilities for increased surveillance of emerging AR organisms to support clinical screenings and future response capabilities. However, the survey needs to be administered more widely and systematically to have an increased resolution of national capacity.

The aforementioned learned lessons and feasibility assessment described are pathogen-agnostic and applicable for generic WWS at post-acute/long-term care facilities. However, key considerations were developed with the intentional application of determining the utility of WWS at healthcare facilities for AR organisms that are commonly associated with healthcare infections and are outlined in Box 1. The goal of correlating AR prevalence from WWS data to the prevalence of AR from patient screening or clinical culture data would be to allow WWS to reduce the need for data from other sources.

### Key considerations: WWS for AR organisms (Box 1)

A vital primary step, combined with the knowledge gained from a tracer study and a feasibility assessment, should emphasize alignment of WW with epidemiological and correlation components. It is critical that facility-level epidemiological metrics are collected to understand the dynamics of the population contributing to the WW signal. These epidemiological data include, but are not limited to, current prevalence of targeted AR organisms (e.g., carbapenemase-producing organisms, C. auris), the number of patients/residents who use the toilet and bathe/shower, and waste management practices (i.e., facility methods for disposing of fecal waste from bed pans, briefs, and bed baths). Communication includes consistent messaging in emails to recruit facilities, provide informational study flyers, signage on field equipment, and monthly reports to the facility (Supplemental 1). An additional consideration for communication materials is to have one designated POC at the facility to manage WWS-related communications. It is recommended that stakeholders (e.g., long-term care facility, WW experts, health departments) take the time to draft a plan that outlines a course of action for each type of WWS result, noting that response plans are specific to the target. Public health has an important role in understanding and responding to AR, and when designing WWS at healthcare facilities, consulting with local and state authorities is recommended due to their knowledge and trusted relationships with the LTACHs/SNFs, facilities at risk for the target organism, and where identifying the target organisms may inform prevention and response activities. Once all stakeholders have committed to engagement in a healthcare WWS effort, written agreements with defined roles and responsibilities are encouraged to establish a clear understanding of expectations, workflows, timelines, data management, results sharing, and response plan.

Alignment of WW samples and point prevalence surveys (PPSs; i.e., rectal swab screening of a ward or facility using verbal consent) from paired same-day collection to result analysis is critical to build evidence toward the utility determination of WWS at healthcare facilities for desired targets. Of note, it is important to utilize a tracer study confirmed onsite WW collection site that verifies the unit where the PPS is being performed. Stakeholders should decide which approach works most effectively, depending on the timing of the PPS and whether screening will be performed across multiple days (e.g., due to large facility size or staffing constraints): WW sampling (a) within a 24- to 48-h window of the PPS or (b) the days of PPS start and completion. Another consideration is understanding how the facility ascertains prevalence and their approaches to assess transmission in an outbreak setting, to allow for coordination with the WW group to conduct additional sampling and/or share aggregate clinical data. A higher frequency of WW sampling, in addition to the paired sampling (WW and PPSs), is also proposed to adequately track the WW background signal. As mentioned, this is a nascent field, and assessing when a correlation determination is finalized per AR target, facility type, geographic region, etc., should be an ongoing conversation among stakeholders.

Assays used for target detection to evaluate the alignment between WW and PPSs and/or patient screenings are also important. A systematic review by Hassoun-Kheir et al. (2020) highlighted that in 37 studies, the heterogeneity of methods applied hampered a comparison of the results (Hassoun-Kheir et al. 2020). Open discussions should occur between the WW and public health labs regarding laboratory assays used and coordination for the WW laboratory to evaluate detection assays using the WW matrix for testing (i.e., concentrate, extract, and/or lysates). Full characterization and validation of assays with positive controls in WW matrices using consistent performance criteria and optimization processes to understand variation in laboratory performance, limit of detection, limit of quantification, recovery efficiency across sites, etc., will improve AR organism assays for WWS. Ideally, inter- and intra-laboratory comparisons using the same primer/probe sequences and detection platforms would be performed for confidence in the WW results to allow more fluent, comparable analysis in determining the utility of healthcare WWS.

## CONCLUSIONS

Publications acknowledge that facility-level AR WWS would likely be more actionable, particularly for AR organisms for vulnerable populations, than community-level WWS, but this has not been robustly tested (NAS 2024; Hassoun-Kheir et al. 2025); one could ascertain this would apply to additional public health targets at a healthcare facility. A main goal with the information presented is to assure that as other programs initiate WWS at healthcare facilities, there are foundational considerations to reference. A narrative review also discussing how to establish a healthcare WWS program found similar conclusions as presented here (Hassoun-Kheir et al. 2025), and together, stakeholders now have a similar, consolidated perspective from two different regions of the world. For the utility determination and potential future use of healthcare WWS, these key considerations bolster the analysis of the correlation between WW data and clinical screening data. Additionally, if programs include these key considerations in a standardized approach, then comparing across programs will be feasible and enhance the ability to determine successful WWS approaches at healthcare facilities. The existence of integrated, public WW working groups that include academic, government, and private sectors (e.g., Association of Public Health Laboratories Community of Practices, Water Environment Federation WW summits) provides an avenue to unify healthcare WWS efforts. While challenges remain, such as addressing the confounding factor of microbial growth and biofilms in the sewer plumbing, broadening use of national feasibility surveys for response, and supporting community outreach to healthcare facilities with hesitancy to participate, WWS continues to hold promise for improving knowledge regarding our vulnerable population’s public health.

## Supplementary Material

Supplement2

Supplement1

Supplement3

## Figures and Tables

**Table 1 ∣:** Healthcare facility types and provided care for in the lessons learned assessment (Facilities A, F, and G; *n* = 3) and site surveys and feasibility determination (Facilities A to P; *n* = 16), noting the following abbreviations: SNF, skilled nursing facility; vSNF, SNF with ventilator capabilities; ND, no data; LTACH, long-term acute care hospital; RH, rehabilitation hospital

Facility ID	Facility type	Provided care
A^a,b^	SNF	Skilled nursing, wound/ostomy, indwelling medical devices^c^
B, C	SNF	ND
D, F^a^	SNF	Skilled nursing, inpatient rehabilitation, wound/ostomy, indwelling medical devices^c^
E	vSNF	Skilled nursing, inpatient rehabilitation, wound/ostomy, ventilator care, indwelling medical devices^c^
G^a^	SNF	Skilled nursing, inpatient rehabilitation, wound/ostomy, indwelling medical devices^c^, tracheostomy
H, I, J, K, L	LTACH	Long-term care, ventilator care
M	RH	Skilled nursing, inpatient and outpatient rehabilitation, wound/ostomy, indwelling medical devices^c^
N	RH & vSNF	Skilled nursing, wound/ostomy, indwelling medical devices^c^
O	Mixed-use	Skilled nursing, memory care, assisted living, and independent living units
P	Hospital	Emergency care, critical care, advanced surgical procedures, pain management, wound care

a Facilities participating in the tracer study.

b Facility participating in low-flow strainer assessment.

c Indwelling medical devices: central vascular catheter, urinary catheter, etc.

**Table 2 ∣:** Tracer studies detection and dissipation time-points at three Georgia skilled nursing facilities

	Visual dye tracer			Viral tracer (*n* = 3 per facility)	
	*n*	Detection	Dissipation	Peak detection	Dissipation
Facility A^a^	2	2.5 min	7 min	2–10 min	40 min
5th floor				(1.35 × 10^3^ to 1.13 × 10^2^ gc/μl)	
Facility F^b^	3	2.0 min	5.5 min	2–15 min	90 min
1st floor				(2.58 × 12^2^ to 1.57 × 10^2^ gc/μl)	
Facility G^c^	1	1.5 min	4.0 min	2–10 min	240 min
1st floor				(6.93 × 10^4^ to 4.18 × 10^2^ gc/μl)	

The visual dye study used the fluorescent tracer dye (Bright Dyes^®^ FLT yellow/green tablet; Kingscote Chemicals, Miamisburg, OH) and the biological tracer studies used heat-inactivated Inforce^®^ 3 Respiratory Vaccine lyophilized calf vaccine (Parsippany, New Jersey; Zoetis) with bovine respiratory syncytial virus (BRSV) as the detected target. Noted are the floor the tracers were introduced into the toilets, detection and peak detection (min), as well as the dissipation time (min).

a Facility A sampling time-points for the viral tracer: 0, 1, 2, 3, 4, 5, 6, 8, 10, 15, 30, 35, 40, 45, 60, 90, and 120 min.

b Facility F sampling time-points for the viral tracer: 0, 1, 2, 3, 4, 5, 6, 8, 10, 12, 15, 30, 45, 60, 90, and 120 min.

c Facility G sampling time-points for the viral tracer: 0, 1, 2, 3, 4, 5, 6, 8, 10, 30, 45, 60, 120, 150, 180, 240, 720, 1,080, and 1,440 min.

**Table 3 ∣:** Pilot survey results at seven Georgia (USA) post-acute care facilities to understand potential sampling locations conditions and any safety concerns for sampling wastewater

Facility	A	B	C	D	E	F	G
Resident census N	169	107	99	61	109	118	92
Number of residents contributing fecal material to wastewater *n* (%)	66 (40%)	16 (15%)	ND	20 (33%)	21 (19%) Approx. 85%		Approx. 60–70%
Manhole appropriate for sampling?	Yes	Yes^a^	Yes^a,b^	Yes	Yes^a^	Yes	Yes
Wastewater flow adequate for sampling?	Yes	Yes	No^c^	Yes	Yes	Yes	Yes
Cleanout accessible for sampling?	Yes	No^d^	No^d^	Yes	No^d^	No^e^	ND
Facility amenable to project?	Yes	Yes	Yes	ND^f^	Yes	Yes	Yes
Open communication avenues at facility?	Yes	Yes	Yes	ND^f^	Yes	Yes	Yes
Safe sampling site?	Yes	Yes	No^g^	No^h^	No^i^	Yes	Yes
Overall, is sampling wastewater feasible at this facility?	Yes	Yes	No	No	No	Yes	Yes

**Abbreviations:** ND, not determined. Approx., Approximately.

a No nearby electrical outlet for autosampler.

b Large heavy square manhole covering was difficult to open with standard manhole opening tools.

c Low wastewater flow in manhole. A sludge mat several inches thick was covering the flow and would require a plumbing intervention to remove debris.

d No cleanout available or unable to locate cleanout.

e Cleanout located in the middle of the dining room.

f Not assessed because sampling site was unsafe and therefore sampling could not be performed at this facility.

g Out of the way of pedestrian and vehicular traffic but the non-standard square manhole covering could pose a safety risk.

h Both manholes on the property were in unsafe locations. One was near the entrance of the facility and the other was close to a street with vehicular traffic.

i Effluent pipe was broken about two feet below the manhole opening which created a safety concern for wastewater splashing out of manhole.

**Table 4 ∣:** An excerpt of expanded pilot survey results at a total of nine post-acute care facilities in Illinois (IL), Utah (UT), Texas (TX), and Arizona (AZ) (USA) focusing on the feasibility for wastewater sampling. See the supplemental 3 for the entire expanded survey questions

Facility	H	I	J	K	L	M	N	O	P
State	IL	IL	IL	IL	IL	UT	TX	AZ	AZ
Resident census	59	98	80	28	30	45	85	11	120
Amenable to WW equipment?	Yes	Yes	Yes	Yes	Yes	Yes	Yes	Yes	Yes
Ameneable to informational signs and fliers?	Yes	Yes	Yes	Yes	Yes	Yes	Yes	Yes	Yes
Is there at least one external physical access point to the WW?	Yes^a^	Yes^b^	No	Yes^a^	Yes^a^	Yes^a^	Yes^c^	Yes	Yes
Where is the external WW access point located?	On land-scaped grounds	On land-scaped grounds	n/a^d^	Public sidewalk	On land-scaped grounds	By dump-sters at loading dock, away from traffic	CO at end of East wing; MH in roadway of parking lot in back of facility	On land-scaped grounds	In parking lot, but where there is traffic
Approx. how far from the building is the WW access point?	20–50 ft	<20 ft	n/a^d^	<20 ft	>100 ft	20 ft	6 ft, 40 ft	20–50 ft	<20 ft
Manhole cover	No	Yes	n/a^d^	No	No	No	No	No	No
a. Energy source/outlet?									
b. MH cover diameter (in)	22.75	22.75	n/a^d^	22.75	22.75	24	24	30	24
Depth (ft)	13	20		4	13	11	11	8	6
Sufficient space for setting up equipment?	Yes	Yes	n/a^d^	Yes	Yes	Yes^e^	Yes^e,f^	Yes	Yes
a. Autosampler b. Passive	Yes	Yes	n/a^d^	Yes	Yes	Yes^e^	Yes^e,f^	Yes	Yes
Any feasibility concerns?	No	No	Yes^d^	Yes^g^	No	Yes^h^	Yes^i^	Pedestrian traffic^j^, commingled flow^k^	Parking lot and daily traffic
Overall, is sampling wastewater feasible at this facility?	Yes	Yes	No	Yes	Yes	Yes	Yes	Yes	Yes

**Abbreviations:** WW, wastewater; CO, cleanout; MH, manhole; n/a, not applicable; Approx., Approximately.

a Access point is a manhole.

b Access point is a manhole lift station.

c One manhole and one cleanout.

d No external physical access point at this facility.

e There is room for sampling equipment if it is placed in the manhole.

f There is room for sampling equipment next to the building near the cleanout.

g Location is in the middle of a public sidewalk.

h Sampling equipment could obstruct garbage pickup location.

i Manhole is in parking lot therefore would need a vehicular traffic diversion. Area could flood with heavy rain.

j Barricades must be used at all times due to pedestrian traffic.

k Two wastewater drainage pipes merge and concerted effort must occur to make sure passive and active sampling is within the comingled flow.

## Data Availability

All relevant data are included in the paper or its Supplementary Information.

## HIGHLIGHTS

- Low-flow sampling challenges and the importance of conducting dye tracer studies are presented.
- A facility wastewater access survey defined characteristics to determine feasibility, which was positively determined for 75% (12 of 16) of surveyed healthcare facilities.
- Correlating wastewater and clinical case surveillance is imperative to determine the utility of healthcare wastewater surveillance.

## Box 1 ∣ Key considerations for healthcare facility wastewater surveillance.

**Table:**

Facility and wastewater sampling location	
Preferred facility type	High acuity post-acute care facility, LTACH^a^ and/or vSNF^b^, where wastewater surveillance can be implemented for greatest impact, depending on the target; and ideally, a facility actively participating in an MDRO^c^ prevention plan with PPS^d^ collection led by a health department.
Site survey	Used to determine the feasibility and safety of wastewater sample collection; wastewater must be collected on the property downstream of the facility and upstream of the introduction of other sources^e^.
Tracer study	This will confirm that the wastewater collection site is capturing the entirety of the intended resident/patient population; a visual dye tracer study is recommended at a minimum.
Implementation	
Epidemiological data	Coordinate with the facility and the jurisdiction health department to collect epidemiological data and facility metadata, including but not limited to case surveillance, quarterly PPSs, and resident/patient contribution to the wastewater, such as toileting and showering data.
Communication	Develop templates for clear, consistent messaging regarding facility recruitment, informational memos, field signs, and results reporting with a designated POC^f^; as well as outline a draft response plan and hold routine calls/meetings with stakeholders.
Roles/responsibilities	Establish clear roles and responsibilities for willing stakeholders with documented agreement of participation and capacity should include, at minimum, long-term care facility, health department of the facility jurisdiction (laboratory and epidemiology), wastewater experts^g^.
Wastewater and case surveillance	
Wastewater	Determine the frequency of collection to understand background community signals while considering capacity for long-term sustainable collection and processing.
Case surveillance	Understand the facility screening practices, where PPSs are performed at least quarterly as part of an MDRO prevention plan, and understand if additional screening is conducted (e.g., admission screening, facility-wide or unit/RF-based^h^ screening; additionally, plan to collect clinical cases of targeted organisms.
Paired samples	Wastewater should be collected within the brief window of time of the PPS being conducted (e.g., within 24 h of the conclusion of a PPS, or before and after the PPS) on a quarterly basis per year.
Assays	The same primer sequences for the target gene/organism should be used in molecular assays, and ideally, the same assay for the wastewater concentrate/extract/lysate and PPS swab (or stool) that have been validated/verified and characterized for wastewater to understand and communicate the limitations for wastewater matrices.

a LTACH: long-term acute care hospital.

b vSNF: Ventilator capable skilled nursing facility.

c MDRO: multidrug-resistant organism.

d PPS: point prevalence survey, colonization screening performed unit- or facility-wide following the identification of a patient/resident with a novel or targeted MRDO, or for purposes of prevention.

e Site survey in Supplemental 2.

f POC: point-of-contact.

g Wastewater experts defined: involvement in an active or previous WWS program, existing capacity to collect WW, laboratory capacity using appropriately validated concentration, extraction, and detection for designated AR targets, authors on a peer-reviewed publication, dashboard, or participation in the National Wastewater Surveillance System or similar programmatic efforts.

h RF-based: risk factor-based.
