# Supplementary material for: Healthcare wastewater surveillance: methodological considerations for sampling, feasibility, and implementation
Source: J Water Health. Author manuscript; Available in PMC 2026 Apr 6. (PMC13051650; doi:10.2166/wh.2025.167)
Supplement: Supplement2 [file NIHMS2156864-supplement-Supplement2.pdf]

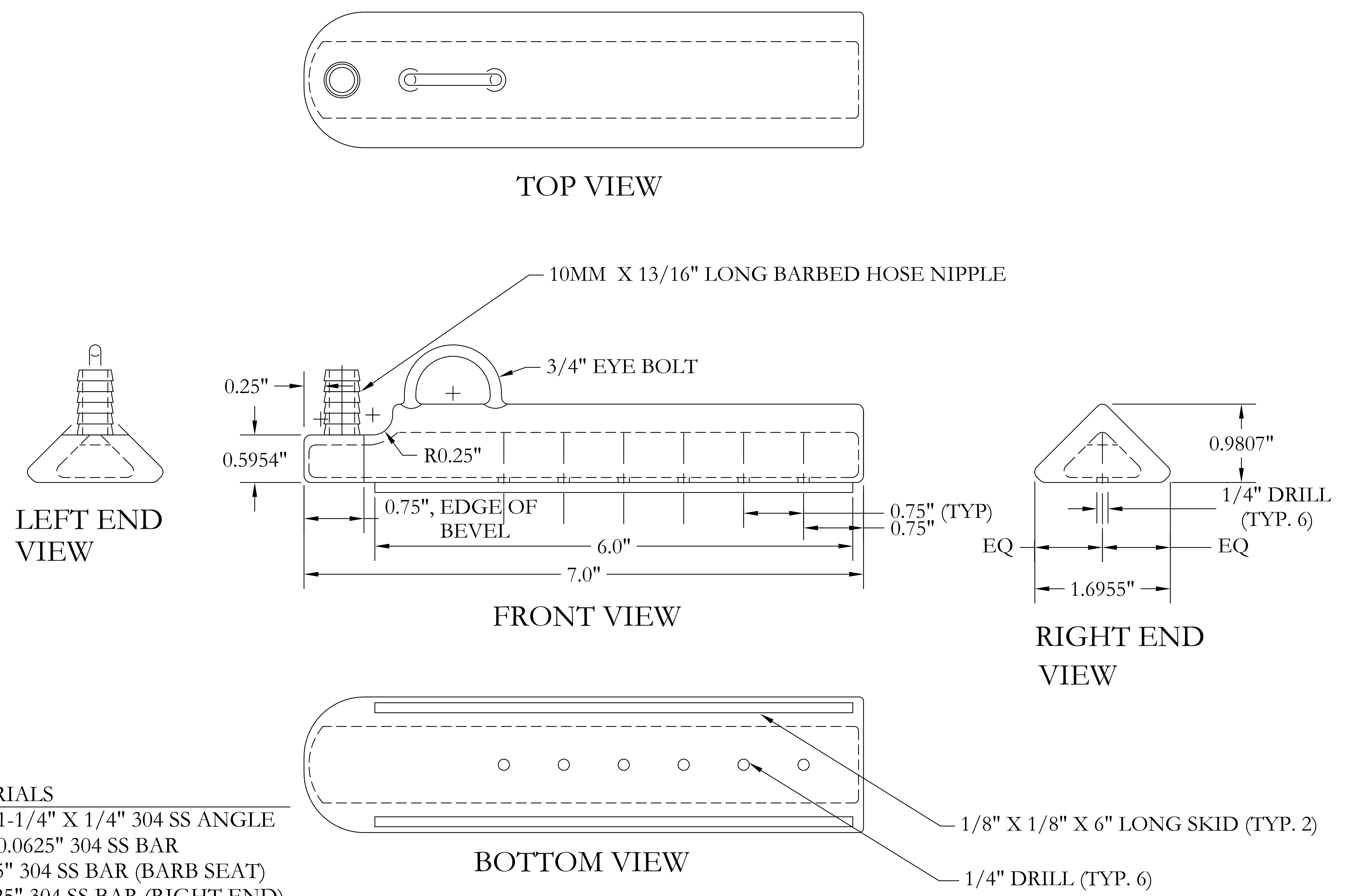

BILL OF MATERIALS

7"	1-1/4" X 1-1/4" X 1/4" 304 SS ANGLE
7"	1-1/2" X 0.0625" 304 SS BAR
3"	1" X 0.125" 304 SS BAR (BARB SEAT)
1-1/2"	1" X 0.0625" 304 SS BAR (RIGHT END)
2-1/2"	3/4" X 0.0625 304 SS BAR (NOSE)
13/16" X 10 MM	304 SS BARBED NIPPLE
3/4" 304 SS U-BOLT	
6"	1/8" X 1/8" 304 SS BAR

REV | DATE | BY

SEAL

U.S. DEPARTMENT OF HEALTH & HUMAN SERVICES
PUBLIC HEALTH SERVICE
CENTERS FOR DISEASE CONTROL AND PREVENTION

SIPHON

DESCRIPTION

Designed by D. Neal Whaley Jr and Christine Ganim;
Technical dawing by Harris Sheinman

PROJECT #: N/A
DATE: 2/13/2023
PROJ. OFFICER: N/A
DRAWN BY: LYY9
CHECKED BY: DSW4
DRAWING #: 1 OF: 1

CONTROLLED
UNCLASSIFIED INFORMATION (CUI)
UNAUTHORIZED DISCLOSURE IS STRICTLY PROHIBITED.
ENHANCED SAFEGUARDING AND DISPOSAL MEASURES ARE REQUIRED.

DWG-001
