# Supplementary material for: Healthcare wastewater surveillance: methodological considerations for sampling, feasibility, and implementation
Source: J Water Health. Author manuscript; Available in PMC 2026 Apr 6. (PMC13051650; doi:10.2166/wh.2025.167)
Supplement: Supplement1 [file NIHMS2156864-supplement-Supplement1.docx]

**Supplemental 1: Methods, Feasibility, and Communication**

1. **Methods**

State partnerships and facility selection

Tracer studies

Strainer assessment, including Supplemental Figure 1. Strainer types

1. **Feasibility individual state results**

Pilot survey: Georgia

Expanded survey: Illinois, Utah, Texas, Arizona

1. **Communication document templates**

Email template

Project summary

Notification letter

Equipment sign

1. **Methods**

State partnerships and facility selection

The Healthcare-Wastewater Antimicrobial Resistant Network (H-WARN) program within the Division of Healthcare Quality Promotion at the CDC (Atlanta, GA) collaborated with the Georgia (GA) Department of Public Health (GDPH). The GDPH provided recommendations and initial communications with healthcare facilities via email. Once positive interest was indicated by healthcare facilities, H-WARN then led communications regarding the WWS effort to recruit for participation over virtual meetings and subsequent in-person site visits. The GA facilities were identified as Facility A to G.

WWS efforts in Illinois (IL) were led by Rush University Medical Center in partnership with University of Illinois Chicago and local public health departments (Chicago DPH and Cook County DPH). A convenience sample of LTACHs was identified through contacts known through prior and existing quality improvement collaborations. The IL facilities were identified as Facility H to L.

WWS efforts in Utah (UT) and Texas (TX) were led by the University of Utah. Contacts from the state health department and lab, individuals involved with statewide HAI efforts and contacts working for target facilities provided recommendations. These facilities were contacted, and initial and follow-up meetings were conducted in person or remotely. Several meetings were required to convince facilities to participate. The UT and TX facilities were identified as Facility M and N, respectively.

WWS efforts in Arizona were facilitated through a combined effort by Arizona State University (ASU); LeadingAge Arizona, a non-profit that advocates on issues of housing, healthcare, and home services for people 65+ years of age; and the City of Tempe Office of Strategic Management and Innovation that oversees wastewater surveillance activities within the community. The AZ facilities were identified as Facility O and P.

Tracer studies

A two-part tracer study, consisted of a visual dye tracer followed by a biological surrogate on a subsequent day, was used to gauge wastewater residence time in pipes after toilet flush and confirm the selected manholes captured the intended population at Facility A, F, and G.  First, a fluorescent tracer dye (Bright Dyes^®^ FLT yellow/green tablet; Kingscote Chemicals, Miamisburg, OH) was flushed from ≤ 2 toilets inside the facility at different floors and wings, and a stopwatch was used to monitor the time taken to visualize the dye.  Time data from the visual tracer was used to establish collection timeframes for the biological tracer study (Table 2).  The biological tracer consisted of a heat-inactivated (60±2°C for 40 min) Inforce^®^ 3 Respiratory Vaccine lyophilized calf vaccine (Parsippany, New Jersey; Zoetis) at an eluted final concentration of 10^6^ to 10^10^ copies per 50 mL buffer, where bovine respiratory syncytial virus (BRSV) was the target biological tracer. A virus surrogate was chosen out of convenience due to an on-going respiratory wastewater surveillance project. The biological tracer study (n=3 per facility) collected wastewater starting at time t_0_ and time points were adjusted between study runs to capture resolution in the early peak and signal dissipation (Table 2). To detect the biological tracer, wastewater samples were concentrated and analyzed using ddPCR (Santiago et al. 2025). Water bills were shared to the CDC team by the facility in the months preceding the tracer study (Facility A, n=9 months; Facility F, n=8 months; Facility G, n=8 months). The average, minimum, and maximum gal per day were calculated as the estimated water use.

The Chicago dye tracer study methods was similar, and more details can be found in their respective publications (Poretsky et al. 2025).

Strainer assessment

Three strainers compatible for use with the Teledyne ISCO Avalanche Autosampler (Lincoln, NE) were evaluated at Facility A with the aim of consistent collection of the desired wastewater sample volume (Figure 1). The Standard Weighted Polypropylene Strainer (“Standard”, n=9; Lincoln, NE; Teledyne ISCO), Low Flow Stainless Steel Strainer (“Low Flow”, n=7; Lincoln, NE; Teledyne ISCO), and a custom fabricated strainer (“Custom”, n=9; Atlanta, GA; CDC) were deployed, and ‘expected’ and ‘collected’ sample volumes were recorded. See Figure 1 for strainer visuals and design differences, as well as and the Custom strainer technical drawing in Supplemental Materials 1 (S1). The ‘expected’ volumes were based upon entered program settings, while ‘collected’ volumes were measured in the field with graduated sample containers. The sample sizes per strainer were not uniform due to (1) evaluation of one strainer at a time, and a strainer design being removed from evaluation when either (2) clogging continuously disrupted sampling, and/or (3) the ‘expected’ versus ‘collected’ were continuously different. Statistical analysis and figure creation used GraphPad version 10.2.0 (Boston, MA), where the absolute values of the ‘expected’ and ‘collected’ differences were used to determine the significance between the groups with Kruskal-Wallis and Dunn’s post-hoc tests (p<0.05).

Figure 1. Strainer types, (a) Standard (photo: https://store.teledyneisco.com) – standard weighted polypropylene strainer (3/8” ID); (b) Low Flow (photo: https://store.teledyneisco.com) – low flow stainless steel strainer (3/8” ID); and (c) CDC custom


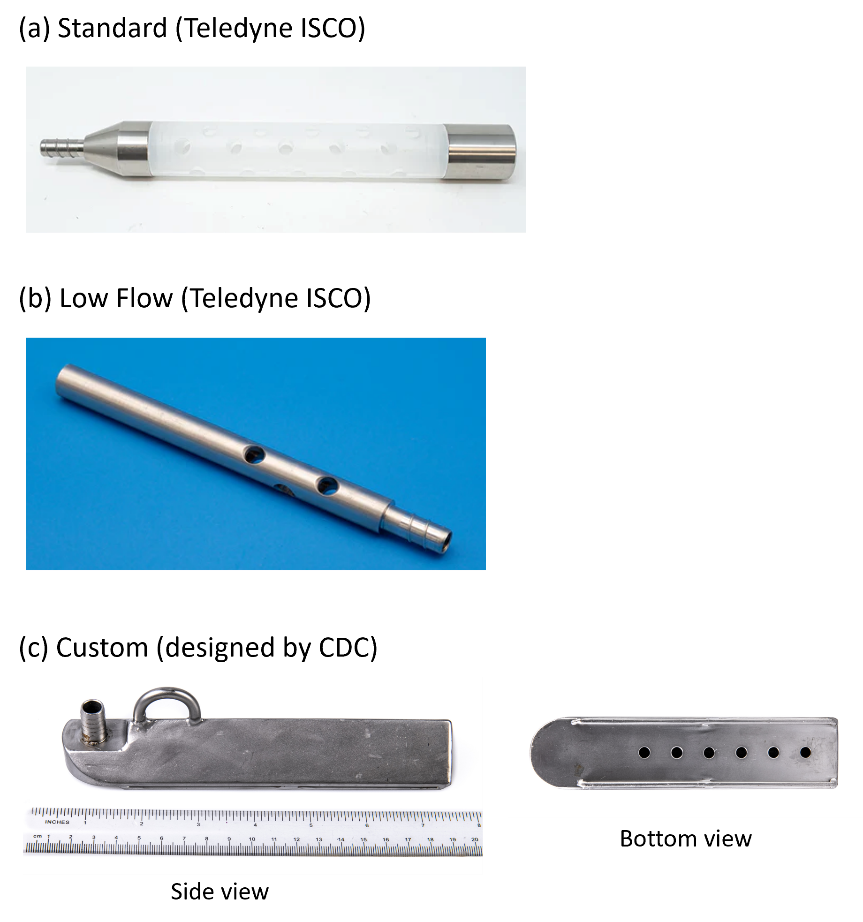


1. **Feasibility – Individual state results**

Pilot Survey

In the GA (Facilities A to G) pilot survey, all seven participating facilities had a manhole identified for potential sampling (wastewater access availability) and while one facility had an unsafe wastewater collection site, all were amenable to the project and open to communication avenues (i.e., handouts to residents/patients to explain project; Table 3). WWS was deemed feasible at 4 (57.1%) of the 7 GA SNFs.

Expanded Pilot Survey

The expanded pilot survey results regarding general wastewater access, wastewater access location, and facility administration aspects, as well as feasibility concerns, are provided in Table 4, where footnotes provide specifics of access point, safety concerns, and extenuating circumstances that would make WWS not feasible. See S3 for the full expanded survey questions, as only highlighted results are included in the text and Table 4.

*Illinois (IL; Facilities H to L)*: Four of the five LTACHs had external physical access to wastewater (three via a single manhole; one via a combination of manhole and wastewater lift station) that captured all the facilities’ flow. These four LTACHs had a manhole location with adequate space for setting up equipment, and one also had an outlet nearby (<20 ft) to power an autosampler. The one facility (Facility J) with no manhole located on the property was not included in any other components of the survey. WWS was deemed feasible at 4 (80.0%) of 5 IL LTACHs.

*Utah (UT; Facility M)*: The RH had external physical access to wastewater via a manhole that captured all the facility wastewater effluent. There was inadequate space to set-up equipment by the manhole due to being located by dumpsters at a loading dock, nor an outlet nearby to power an autosampler. However, there was adequate space within the manhole to deploy an autosampler. WWS was deemed feasible at the UT RH.

*Texas (TX; Facility N)*: The vSNF had external physical access to wastewater via a manhole that captured all the facility wastewater effluent. There was not adequate space to set-up equipment by the manhole, which was in a parking lot behind the facility, adjacent to an access road; and no proximal outlet to power an autosampler was available. However, there was adequate space within the manhole to deploy an autosampler. WWS was deemed feasible at the TX vSNF.

*Arizona (Facilities O and P)*: Both facilities (mixed-use and hospital) had external physical access to wastewater via a manhole that captured all the facility wastewater effluent. The location of the manhole at both facilities had adequate space for setting up equipment, but there were no outlets nearby for either location to power an autosampler. One of the facilities had pedestrian traffic near the manhole, thus a notable safety concern during sampler deployment/retrieval and the other was located in a parking lot within an accessible parking spot requiring planning to ensure the parking space was empty. WWS feasibility was positively determined at both facilities (2 of 2, 100%).

1. **Communication document templates**

The following communication documents are templates to aid in reaching out to facilities with the assistance of your local health department: (1) email – recommend that the local health department use this email to reach out to facility to gauge initial interest; (2) project summary – briefly describes the wastewater surveillance effort and can be attached to the initial email or sent after the facility confirms interest; (3) notification letter – a letter than can be prepared to email to staff and families, as well as to have hard copies at the front desk for distribution; and (4) signs for visible equipment on the property – signs to direct any questions to the entity conducting wastewater surveillance versus the facility staff, and noting that having hard copies of the notification letter at the front desk is also in preparation for questions that may arise from the staff, public, or families regarding the equipment. Two additional considerations are to prepare a standardized report and establish a frequency at which the facility, if amenable, would like to receive results; and finally, prepare a “Certificate of Recognition” to acknowledge the facilities participation as a gesture at the end of the surveillance effort.

1. Email

*Good morning [Insert Facility Administrator],*

*I am with the [your group name here]. We have been working with the [add collaborators here, especially and state department of health] and we thought your facility may be a good site for public health surveillance.*

*To provide some background, we would like to sample Long-Term Care Facility wastewater to see if our methods are able to detect antimicrobial-resistant organisms in wastewater as a potential early detection system.*

*We understand this is another extremely challenging time for facilities.*

*This public health surveillance does* ***not*** *involve any direct contact with residents and would not require us to go into the facility, but rather we would be sampling wastewater from [location here] and request minimal general information a [frequency - day/week] per [frequency - month/quarter] that could be answered over the phone or email.*

*We were hoping the [Facility Name Here] would be interested in participating in this public health surveillance effort.*

*I have attached our plan, and we were hoping you may be open to a meeting to discuss this further?*

*Thanks so much,*

1. Project summary

***Title: Surveillance of Antimicrobial Resistance (AR) in Long-Term Care Facility Wastewater***

***Project Description:*** *[Your entity here] is planning to perform sampling of wastewater from long-term care facilities (LTCF) to detect antimicrobial resistance (AR) to develop an early warning system for emerging AR pathogens in the coming year(s). We are inquiring about your interest in including your facility in the study.*

*We are seeking long-term care facilities that would allow the [Your entity here] team to:*

1. *Collaborate with your administrator, director of nursing, and infection preventionist to understand the facility AR prevalence, as well as the facility grounds manager to discuss wastewater sampling plans considering impacts of aesthetics, safety, and security.*
2. *Test the wastewater effluent at [Your laboratory here] for AR organisms/genes (number here times a month for duration here). We will regularly report the results of AR testing of wastewater samples to the facility administration, frequency as per the facility and administrations preference.*
3. *Collaborate with the facility and [Your Public Health Department here] to gather epidemiologic and facility data to provide insight about the presence of certain AR pathogens at the facility.*
4. *PPS [Understanding this is state/health department/facility dependent – add reasonable expectations to correlate wastewater data to clinical screening]*
5. *Collaborate with the facility and [Your Public Health Department here] to provide guidance on how to interpret positive signals from wastewater testing. This may lead to recommendations from [Your entity here] or [Your Public Health Department here] for the facility to perform additional resident screening for AR as part of a response at the facility.*

*We are hoping you will collaborate with our Team to validate the utility of wastewater surveillance as a possible solution to identify AR pathogens in Long-Term Care Facilities before outbreaks occur. This project was determined to be public health surveillance by [Your entity here] and not human subjects research [adjust wording depending on your clearances needed].*

***Research Team:*** *This study will be performed by a team of researchers in the [Your entity here, more specifics to as department/division/etc.].*

*Contact information: email(s), phone number(s) of main points-of-contact(s)*

1. Notification letter

*[Letterhead recommended for official recognition]*

*Dear Sir/Madam,*

*Your facility and the [Your Public Health Department here] has approved a team from the [Your entity here] to collect wastewater samples [number of samples per week, frequency] over the next month, and potentially up to [duration here]. You may see representatives from [Your entity here] gathering information about antimicrobial resistance by collecting wastewater samples from a manhole on the facility’s property. The information collected could help us find out whether using wastewater could be a possible way to identify antimicrobial resistance in [facility here] before outbreaks happen.*

*Your facility, [Your Public Health Department here], and [Your entity here] will look at the laboratory information collected from the wastewater and antimicrobial resistance screening data in the facility. The wastewater samples will be collected with [sampling approach here] sampling system, which will be installed in a place that does not affect the safety or security of residents, staff, or visitors. This project does not involve direct contact with residents, and we will not access any personally identifiable information. This project will not have any impact on medical treatments or residents’ ability to receive care.*

*The findings from this project will help us understand whether wastewater surveillance is a possible approach to identify antimicrobial resistance outbreaks in healthcare facilities. The findings may also help reduce the spread of antimicrobial resistance at your facility keeping you and others safe. The [Your entity here] will do their best not to interrupt normal operations and traffic.*

*Thank you for your understanding. If you have any questions, please feel free to email the [Your entity here] team at [email address here].*

*Thank you,*

*The [Your entity here] Team*

*[Your entity here]****:*** *This study will be performed by a team of researchers in the [more details about your entity here, division/department/etc.].*

1. Sign
