# Supplementary material for: Healthcare wastewater surveillance: methodological considerations for sampling, feasibility, and implementation
Source: J Water Health. Author manuscript; Available in PMC 2026 Apr 6. (PMC13051650; doi:10.2166/wh.2025.167)
Supplement: Supplement3 [file NIHMS2156864-supplement-Supplement3.docx]

**Purpose:** To assess wastewater access at healthcare facilities, particularly skilled nursing facilities and long-term acute care hospitals. These data will be used to understand the feasibility of a national wastewater surveillance program at healthcare facilities. This survey includes an assessment of wastewater access points, the facility’s willingness to participate in a wastewater surveillance program, and the safety and feasibility of sampling wastewater at facilities.

| **Section 1: Administrative** | |
| --- | --- |
| **Questions (8)** | **Answer options** |
| 1. Year facility was built (four-digit year) | ________ (four-digit year)  ☐ unknown |
| 1. Number of floors in the facility (number) | ________ floors |
| 1. Number of licensed beds or capacity (number) | ________ licensed beds or capacity |
| 1. Census or average occupancy (number) | ________ patients or residents |
| 1. Is the facility amenable to wastewater sampling equipment being set up long-term (i.e., greater than 6 months)? (choose one) | Y☐ / N ☐ / Unsure ☐ |
| 1. Is the facility open to an informational sign on the wastewater sampling equipment and a flier to provide to the public if they have questions? (choose one) | Y☐ / N ☐ / Unsure ☐ |
| 1. Are staff available daily if issues arise?    1. Grounds (choose one)    2. Plumbing (choose one)    3. Engineering (choose one) | a. Y☐ / N ☐ / Unsure ☐  b. Y☐ / N ☐ / Unsure ☐  c. Y☐ / N ☐ / Unsure ☐ |
| 1. Preferred facility point of contact | Name (text):  Phone number:  Email address: |

| **Section 2: Willingness to participate** | |
| --- | --- |
| **Questions (4)** | **Answer options** |
| 1. How likely would your facility be to participate in a wastewater monitoring program if it could provide useful information about patient/resident antibiotic resistance burden for infection control? (choose one) | On a scale of 1 (Very likely) to 5 (Very unlikely)  1 ☐ Very likely  2 ☐ Likely  3 ☐ Neutral  4 ☐ Unlikely  5 ☐ Very unlikely |
| 1. What would make your facility more likely to participate in a wastewater monitoring program? (text) | Describe (text): |
| 1. What are potential barriers to participating in a wastewater monitoring program at your facility? | Describe (text): |
| 1. If applicable, what is the likelihood that your corporate office would support your facility’s participation in a wastewater monitoring program? (choose one) | On a scale of 1 (Very likely) to 5 (Very unlikely)  1 ☐ Very likely  2 ☐ Likely  3 ☐ Neutral  4 ☐ Unlikely  5 ☐ Very unlikely  ☐ Not applicable |

| **Section 3: Wastewater access** | | |
| --- | --- | --- |
| **Questions (6)** | **Answer options** | |
| 1. Is there at least one external physical access point to the wastewater stream that captures all the facility flow (e.g., sewer manhole on the property)? (choose one) | Y☐ / N ☐ / Unsure ☐  If yes, describe (text): |  |
| 1. Where is/are the external wastewater access point(s) located? (choose all that apply) | ☐ In your parking lot, away from traffic  ☐ In your parking lot, but where there is traffic  ☐ In the street, but not in the lanes of traffic  ☐ In the street and in the lanes of traffic  ☐ In an area designated for emergency vehicles, deliveries, and transportation services  ☐ On the landscaped grounds surrounding/next to your building  ☐ On the sidewalk or in the space between the sidewalk and the street  ☐ In an area with foot traffic (e.g., entrance, visitor walkway, resident patio)?  ☐ Unsure  ☐ Other  Describe the area (text): |  |
| 1. Approximately how far from the building is/are the wastewater access point(s)? (choose all that apply) | ☐ <20 ft  ☐ 20-50 ft  ☐ 50 – 100 ft  ☐ >100 ft  ☐ Don’t know the location of the manhole(s) |  |
| 1. Manhole cover size (diameter) and other characteristics:    1. Energy source/outlet available? (choose one):    2. Description of manhole (e.g., external influences, low point where flooding occurs, near the entrance or parking lot) (text): | a. Y ☐ / N ☐ / Unsure ☐  b. Describe (text): |  |
| 1. Is there sufficient space for setting up the following equipment and for 1 to 3 project staff to work near an appropriate wastewater access point?    - - - 1. Autosampler (choose one)          2. Passive (choose one)          3. Other (choose one) | a. Y☐ / N ☐ / Unsure ☐  b. Y☐ / N ☐ / Unsure ☐  c. Y☐ / N ☐ / Unsure ☐  If yes to 2c, describe (text): |  |
| 1. Assess the sampling location for any feasibility concerns and describe (text): | Describe (text): |  |

| **Section 4: Grease traps/drainage lines** | |
| --- | --- |
| **Questions (7)** | **Answer options** |
| 1. Does your facility have an external or outside grease trap? (choose one) | Y☐ / N ☐ / Unsure ☐ |
| 1. Does your facility have a wastewater drainage line (lateral) to the sewer that is separate from the grease trap? (choose one) | Y☐ / N ☐ / Unsure ☐ |
| 1. Is the sample location intermingled with the flow to or from the grease trap? (choose one) | Y☐ / N ☐ / Unsure ☐ |
| 1. How many wastewater drainage lines (laterals) other than the grease trap leave the building? (number) | ____________(number) |
| *The questions below are for the first drainage line. (Repeat the following questions for the number of drainage lines)* | |
| 1. Is there a clean out that can be used to access the drainage pipe outside the facility that is associated with the manhole? (choose one) | Y☐ / N ☐ / Unsure ☐ |
| 1. Approximate diameter of the clean out (number)    1. Diameter (number)    2. Units of diameter (choose one) | 1. ________ (number) 2. Inches ☐ / Centimeters ☐ |
| 1. Is the clean out horizontal or vertical? (choose one) | ☐ Horizontal  ☐ Vertical  ☐ Unsure |
| *Repeat the section above as needed based on number of drainage lines.* | |

| **Section 5: Plumbing questions** | |
| --- | --- |
| **Questions (3)** | **Answer options** |
| 1. Potable water meter location (text) | Describe (text): |
| 1. Is laundry done onsite? (choose one) | Y☐ / N ☐ / Unsure ☐ |
| 1. Does the facility plumbing system have any bleach (chlorine) injectors installed with the laundry or to treat wastewater before it enters the county sewer system? (choose one) | Y☐ / N ☐ / Unsure ☐  If yes, specify area where installed (text): |

**Definitions**

Bleach injector: Also known as a chlorine injector; the method of adding or mixing chlorine solution into wastewater using an injector pump

Clean out: An access point in the main sewer line for the purposes of cleaning and unclogging the sewer line, installed outside the facility

Grease trap: Trap or waste pipe designed to prevent most greases and solids from entering a wastewater system

Wastewater: Water that has come in contact with human body wastes such as from toilets or other receptacles, as well as water from showers, sinks, drains, etc. This water typically contains pollutants and pathogens that would negatively impact receiving waters unless treated.

Wastewater monitoring: A consistent collection of wastewater and testing of the wastewater for desired targets (e.g., virus, bacteria, genes) over a defined period

Wastewater sampling equipment: Refers to equipment used to collect wastewater and measure characteristics of wastewater; examples include an autosampler and components (e.g., tubing, pump), flow meter, instruments to measure temperature and pH
